# Plasma Amino Acids Reflect Cartilage Loss, Osteoarthritis Pain, Functional Disability, and Mental Health in a Longitudinal Study with Total Knee Replacement

**DOI:** 10.1177/19476035251360189

**Published:** 2025-08-10

**Authors:** Anne-Mari Mustonen, Laura Säisänen, Lauri Karttunen, Petro Julkunen, Amir Esrafilian, Jusa Reijonen, Jussi Mäki, Heikki Kröger, Jari Arokoski, Petteri Nieminen

**Affiliations:** 1 Institute of Biomedicine, School of Medicine, Faculty of Health Sciences, University of Eastern Finland, Kuopio, Finland; 2 Department of Environmental and Biological Sciences, Faculty of Science, Forestry and Technology, University of Eastern Finland, Joensuu, Finland; 3 Department of Clinical Neurophysiology, Kuopio University Hospital, Kuopio, Finland; 4 Department of Technical Physics, Faculty of Science, Forestry and Technology, University of Eastern Finland, Kuopio, Finland; 5 Department of Rehabilitation, Kuopio University Hospital, Kuopio, Finland; 6 Department of Bioengineering, Stanford University, Stanford, CA, USA; 7 Department of Orthopaedics, Traumatology and Hand Surgery, Kuopio University Hospital, Kuopio, Finland; 8 Kuopio Musculoskeletal Research Unit, University of Eastern Finland, Kuopio, Finland; 9 Division of Rehabilitation, Department of Internal Medicine and Rehabilitation, Helsinki University Hospital and University of Helsinki, Helsinki, Finland

**Keywords:** amino acids, functionality, mental health, osteoarthritis, pain, quantitative sensory testing

## Abstract

**Objective:**

Biofluid amino acids (AAs) are potential biomarkers and therapeutic targets for knee osteoarthritis (KOA), a disease continuum of both mechanical and inflammatory aspects. Our aim was to identify AAs that would associate with cartilage degradation, subjectively and objectively assessed joint pain and function, and psychological well-being.

**Design:**

Fasting blood was sampled from 8 healthy controls at baseline, and from 8 end-stage KOA patients before total knee arthroplasty and 1 year post-operatively. Plasma AA profiles were determined with high-performance liquid chromatography, and the obtained results were analyzed with univariate and multivariate statistical tests, and with pathway analysis by MetaboAnalyst.

**Results:**

Cystine, *β*-alanine, and hydroxylysine emerged as potential biomarkers distinguishing KOA patients from controls, and several metabolic pathways were disturbed in baseline KOA. Total knee arthroplasty reduced pain and improved joint function, but the effects on plasma AA metabolism were less obvious. There were significant associations between systemic AA levels and articular cartilage thickness, KOA pain, physical performance, corticospinal excitability, and mental health, independent of age and body adiposity.

**Conclusion:**

The results suggest that AA metabolism could play a role in KOA pathophysiology and motivate further studies investigating the potential of AAs as diagnostic biomarkers and therapeutic targets.

## Introduction

Osteoarthritis (OA) is an age-associated joint disease and one of the leading causes of chronic pain and functional disability. The pooled global prevalence of knee OA (KOA) is 23% in individuals aged 40 and over, and the pooled global incidence is 203 per 10,000 person-years in individuals aged 20 and over.^1^ The burden is expected to continue with the increased life expectancy and aging of the global population.^2^ OA was long considered a “wear and tear” disease leading to the loss of cartilage, but it has subsequently been defined as a condition with multifactorial etiology with both mechanical and inflammatory factors.^3^ The molecular mechanisms underlying the pathogenesis of OA remain inadequately understood, and there are complex relationships between factors, such as overweight, sex, joint loading, local and systemic inflammation, and pain.^4^ Radiographic findings are not associated with pain in a straightforward manner, while synovitis and bone marrow lesions do correlate with pain when using magnetic resonance imaging (MRI). There is no treatment to cure or reverse OA. Only its symptoms can be managed, eventually leading to joint replacement surgery as the only option for severe cases. This is largely because by the time symptoms manifest, the disease has already progressed to a point where cartilage repair becomes unlikely.

OA pain is complex, multifactorial, and modified by genetic, psychological, and environmental factors, and disease-modifying and pain-alleviating therapies are not totally satisfactory.^4^ Patients can display hyperalgesia (i.e., lowered pressure pain thresholds [PPTs] when a force is applied to the joint) and allodynia (non-noxious stimuli perceived as painful). Different pain types, including nociceptive, inflammatory, and neuropathic pain, can overlap in chronic pain conditions, and there is a crucial need for novel biomarkers for clinical use in this field.^5^ Amino acids (AAs) are of special interest because they are not only the fundamental building blocks of enzymes and other proteins but also play essential roles in cellular structures and functions, including pain transmission.^6^ Particular AAs function as excitatory or inhibitory neurotransmitters in the central nervous system (CNS), serve as precursors for neurotransmitters, or have neuromodulatory effects.^7^

Previous studies have demonstrated alterations in the concentrations of AAs and related molecules in body fluids in chronic pain conditions, including OA.^8-11^ For instance, modifications in the profiles and metabolism of arginine (Arg), glutamate (Glu), alanine (Ala), and branched-chain AAs (BCAAs) have been reported. Arg depletion may result from an overactive Arg-to-ornithine (Orn) pathway and lead to an imbalance between cartilage repair and degradation.^11^ Circulating concentrations of Ala, 4-hydroxyproline (HPro),^12^ and BCAAs can increase in KOA^9^ and the ratios of BCAAs to histidine (His) have also been suggested as KOA biomarkers.^13^ As AAs can induce anti-inflammatory and antioxidative effects, they may have potential as immunomodulatory nutrients to treat OA patients.^8^ However, additional data will be necessary to conclusively determine whether the relationships between AAs and OA onset and progression are causal. Moreover, most previous studies did not compare AA concentrations to perceived symptoms and the OA patients’ coping with the disease. For the autoimmune-driven rheumatoid arthritis (RA), significant inverse correlations were reported between the visual analog scale (VAS) global well-being and plasma levels of Ala, Arg, Glu, asparagine (Asn), His, proline (Pro), and serine (Ser), as well as between the number of tender joints and Glu and His levels.^14^ Evidence from animal experiments suggests that exogenous administration of Glu with aspartate (Asp) into the joint space can initiate hyperalgesia and allodynia, while administration of excitatory AA receptor antagonists suppresses the increase in nociceptive behavioral responsiveness.^15^

Early-stage OA is a challenging research topic because it is often asymptomatic or presents with minimal symptoms, yet it can progress into a debilitating joint disease. The sampling of synovial fluid (SF) or joint tissues during asymptomatic early-stage OA would be invasive, associated with an increased risk of infections and, thus, considered unethical. At present, there are no established biochemical or genetic sets of biomarkers of clinical utility that would reliably predict the onset or progression of OA.^9^ Previous literature suggests that AAs could have potential as biomarkers to diagnose OA, assess disease progression, and serve as therapeutic targets for OA pathogenesis.^8-10^ The aim of the present study was to identify novel AA biomarkers that would correlate with cartilage degradation and subjectively and objectively assessing joint pain and function in KOA. As blood metabolite composition can indirectly reflect biological processes in the joint and blood is more accessible than SF or synovial tissues, plasma was the biofluid of choice for the present study. We hypothesized that (1) the presence of KOA would be reflected in plasma AAs compared with healthy controls, and (2) plasma AA profiles would be associated with pain perception and joint function before and after function restorative (i.e., joint replacement) surgery.

## Material and Methods

### Ethics and Subjects

**Figure 1.**
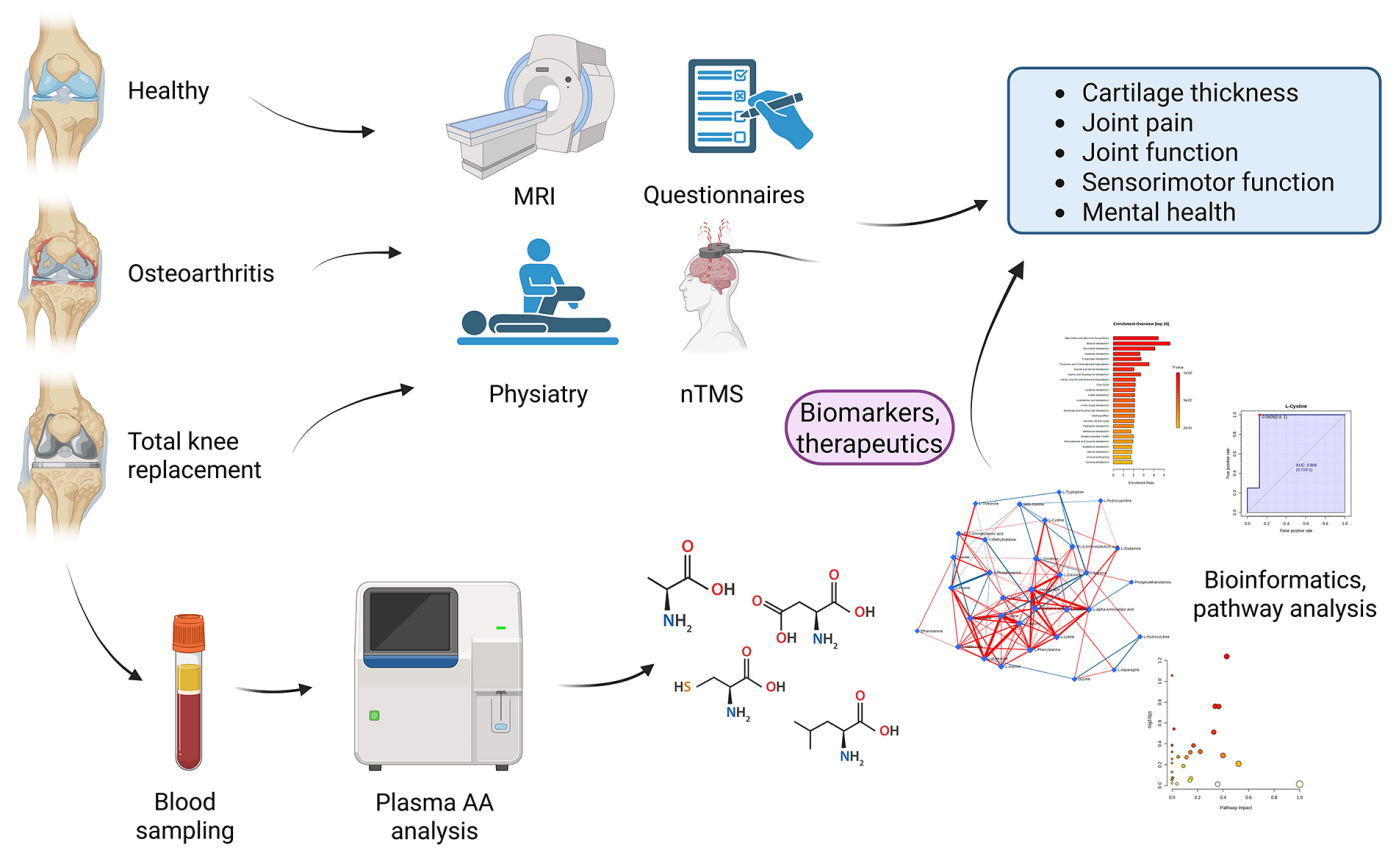
Illustration summarizing the study protocol. AA = amino acid; MRI = magnetic resonance imaging; nTMS = navigated transcranial magnetic stimulation. Created with BioRender.com, Mustonen (2025), https://BioRender.com/q85r620.

This study was approved by the Ethical Committee of Kuopio University Hospital (#140/2017, amended 8/2020) in accordance with the Helsinki Declaration. The privacy rights of human subjects were observed, and all subjects provided written informed consent to donate their blood samples for research purposes. The study protocol has been summarized in **Figure 1**. The subjects (n = 2 men, 6 women) were total knee arthroplasty (TKA) patients with end-stage primary KOA, who were recruited and operated on at Kuopio University Hospital between 2020 and 2022. Healthy joint disease-free controls (n = 4 men, 4 women) were recruited from the staff of Kuopio University Hospital. Inclusion criteria for the patients were as follows: TKA as the indication; age 45–70 years; moderate to severe radiographically verified KOA; and pain of the tibiofemoral joint on most days. Patients were excluded if they had severe pain, limited range of motion (ROM), or substantial instability of the knee caused by other diseases; radiologically determined too mild or too advanced KOA; neurological or metabolic diseases, active malignancies, inflammatory arthritis, earlier restorative surgery of the knee; metal objects or implants in the body if not compatible with MRI scanners, cardiac pacemaker; body mass index (BMI) >33 kg/m^2^; and thigh circumference >52 cm. Gender, age, body mass, height, BMI, and medication were recorded.

### Sampling and AA Analysis

Fasting blood samples were collected from KOA patients before TKA (n = 8) and approximately 12 months post-operatively (n = 8). The controls (n = 8) were only sampled at baseline. Venous blood was collected after overnight fasting using BD Vacutainer K2 EDTA tubes (Becton, Dickinson and Company, Franklin Lakes, NJ). The samples were centrifuged at 2500 × *g* for 15 min at room temperature, followed by a similar second centrifugation of the upper layers of the supernatant to remove any remaining platelets. The obtained platelet-poor plasma was aliquoted and stored at −80°C. Plasma AA profiles were analyzed at an accredited laboratory (HUSLAB, Helsinki, Finland) by using the Bio 30+ (Physiological system) high-performance liquid chromatography cation exchange system with ninhydrin detection (Biochrom, Cambridge, UK).

### Cartilage Thickness, Measures of Physical Medicine, and Neuromuscular Function

We assessed correlations between the AA results and other pre- and post-operative data that had been collected during the outpatient visits. We used several health- and pain-related questionnaires, physical performance-based measures, quantitative sensory testing, and neurophysiological examination to assess the functional status of the patients. The collection and analysis of these data have been described in detail previously, with baseline data partially published.^16^

Articular cartilage thicknesses in the principal load-bearing areas of the medial tibia and femur of control and OA knees were determined by MRI (Philips Achieva 3.0T X; Philips, Eindhoven, the Netherlands, or Siemens MAGNETOM Vida; Siemens Healthcare, Erlangen, Germany) with an automated pipeline implemented in Python based on the tissue geometries obtained from a deep-learning segmentation tool, nnU-Net.^17^

Additional self-assessment and physiatric measurements were (1) self-reported questionnaires (Western Ontario and McMaster Universities Osteoarthritis Index (WOMAC),^18^ VAS,^19^ painDETECT,^20^ Pain Self-Efficacy Questionnaire (PSEQ),^21^ RAND-36 measure of health-related quality of life,^22^ Beck Depression Inventory (BDI), and Beck Anxiety Inventory (BAI)^23^); (2) physical performance measures (ROM of the knee joint,^24^ 30-s chair-stand test, 40-m fast-paced walk test, and 12-step stair-climb test recommended by the Osteoarthritis Research Society International);^25^ and (3) quantitative sensory testing (PPT, thermal detection and heat pain thresholds,^26,27^ and two-point discrimination (TPD) threshold).^28^ The questionnaires, except for VAS, were not obtained from the controls.

The neurophysiological resting motor threshold (rMT), motor map (an estimate of the cortical representation area) of the *tibialis anterior* muscle (Map_TA), and long-interval cortical inhibition (LICI) were determined using navigated transcranial magnetic stimulation (nTMS), as described previously.^16^ The measurements were obtained from the cerebral hemisphere contralateral to the OA knee. The LICI-likelihood was determined as the % of motor evoked potentials (MEPs) that were fully inhibited out of 20 trials.

### Statistical Analyses

Statistical analyses were performed with the IBM SPSS Statistics *v*27 software (IBM, Armonk, NY). Basic statistics (mean ± SE) were calculated for all measured parameters. For the anthropometric and physiatric measurements, the comparisons between 3 study groups (control, KOA baseline, KOA 12 months) were performed using the Kruskal–Wallis analysis of variance (ANOVA) and those between 2 study groups (KOA baseline, KOA 12 months) using the Mann–Whitney *U* test. *P* value <0.05 was considered statistically significant. AA concentrations were compared using the generalized linear model (GLM) according to 3 groups and sex. Correlations were calculated with the Spearman correlation coefficient. The univariate ANOVA, adjusted for the potentially confounding factors of age and BMI, was used to investigate the biologically most relevant and statistically significant associations between AA proportions and variables of cartilage degradation, pain, physical performance, and neuromuscular measurements. To analyze how clearly the diagnosis groups and timepoints differed from one another and which variables separated them most clearly, the supervised linear discriminant analysis (LDA) was performed for the individual AAs and related compounds.

### Pathway Analyses

The MetaboAnalyst software *v*6.0, an open resource for metabolomics data analysis (https://www.metaboanalyst.ca), was utilized to identify the key pathways of AAs and related molecules. The values were normalized by median, and the overall data normalization was attained by log transformation. Baseline KOA was compared with control, and post-surgery KOA to baseline KOA. AAs that could have potential as biomarkers were selected according to the variable importance in projection (VIP) values and the loadings plots of the partial least squares discriminant analysis (PLS-DA) model. The diagnostic performance of each biomarker was assessed by calculating the area under the receiver operating characteristic (ROC) curve (AUC) and determining sensitivity and specificity. In addition, the relevance of individual variables in classifying the study groups was assessed by the Random Forest analysis with 1000 trees by computing the mean decrease accuracy.

## Results

### General Variables

The sex ratios did not differ between the study groups (Fisher’s exact test, *P* = 0.608), but the controls were younger and had lower body adiposity compared with the KOA patients (control: 30 ± 2.5 years, 69 ± 2.7 kg, 23.3 ± 0.74 kg/m^2^; KOA: 61 ± 2.2 years, 91 ± 4.3 kg, 32.3 ± 0.52 kg/m^2^; Mann–Whitney *U* test, *P* = 0.001–0.002).

### Physiatric Measurements, Questionnaires, and nTMS Data

The KOA patients had a smaller ROM (angle of flexion, extension) in their affected knee, worse performance in physical function tests, higher self-reported VAS pain scores, lower PPTs on lateral and medial tibial condyles, and higher rMTs compared with the controls (Kruskal–Wallis ANOVA, *P* < 0.001–0.046). Based on VAS, painDETECT, and WOMAC questionnaires at 12 months post-surgery, TKA improved self-reported pain, joint stiffness, and physical limitation scores in 14 out of 17 categories, as well as the total score (Kruskal–Wallis ANOVA, Mann–Whitney *U* test, *P* < 0.001–0.035). However, the angle of flexion, PPT on lateral tibial condyle, and physical function test results remained lower and rMT elevated 12 months after TKA compared with the controls (Kruskal–Wallis ANOVA, *P* < 0.001–0.031). TPD thresholds (lateral and medial knee joint, reference forearm), thermal thresholds (warm, cold, heat pain), PPTs (lateral and medial joint capsules, *rectus femoris* muscle, patella, reference forearm), Map_TA, LICI, LICI-likelihood, and electric field strength did not differ between the 3 study groups, and general health (RAND-36), PSEQ, BDI, and BAI did not differ between the KOA groups at baseline and 12 months post-surgery (Kruskal–Wallis ANOVA, Mann–Whitney *U* test, *P* > 0.05).

### AA Concentrations

A total of 34 AAs and related molecules were detected by the high-performance liquid chromatography cation exchange system. The average AA concentrations and statistical differences between the 3 study groups are represented in Supplemental **Table S1**. Based on the GLM, study group had significant effects on the levels of the following AAs and related compounds: phosphoserine (PSer), phosphoethanolamine (Pea), threonine (Thr), Ser, citrulline (Cit), cystine, methionine (Met), *β*-alanine (Bala), *β*-aminoisobutyric acid (BAIBA), hydroxylysine (HLys), Orn, and 1-methylhistidine (1-MetHis). For instance, the controls had higher Thr, Bala, and 1-MetHis concentrations than the KOA baseline group, and the cystine levels were highest in the post-surgery KOA group. Men had higher concentrations of Pea, glutamine (Gln), Ala, valine (Val), Met, isoleucine (Ile), leucine (Leu), phenylalanine (Phe), Orn, HPro, Pro, BCAAs, essential AAs (EAAs), and total AAs (TAAs), and lower levels of *α*-aminoadipic acid (AAA), glycine (Gly), and 1-MetHis compared with women.

**Figure 2.**
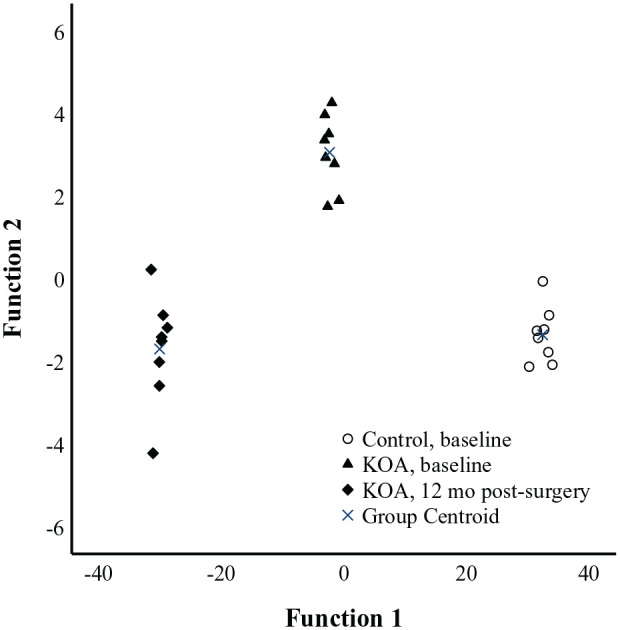
Linear discriminant analysis depicting the classification of plasma amino acid data (μmol/l) in controls and knee osteoarthritis (KOA) patients based on discriminant functions 1 (on the x-axis) and 2 (y-axis) that together explain 100% of the variance in the dataset. White symbols = controls, black symbols = KOA patients.

Using all individual AAs and related compounds as variables, the supervised LDA classified 100% of the samples into their correct study group (**Fig. 2**). The most important contributors to the model were Gly, Met, taurine (Tau), Asp, Bala, Glu, Cit, Ala, Pea, and Asn. Discriminant function 1 (on the x-axis of **Fig. 2**) explained 99.3% of the variance in the dataset and separated all study groups from each other, especially the controls from the KOA patients 12 months post-surgery. Discriminant function 2 (on the y-axis) explained only 0.7% of the variance.

### Associations of AAs to Other Measurements

**Figure 3.**
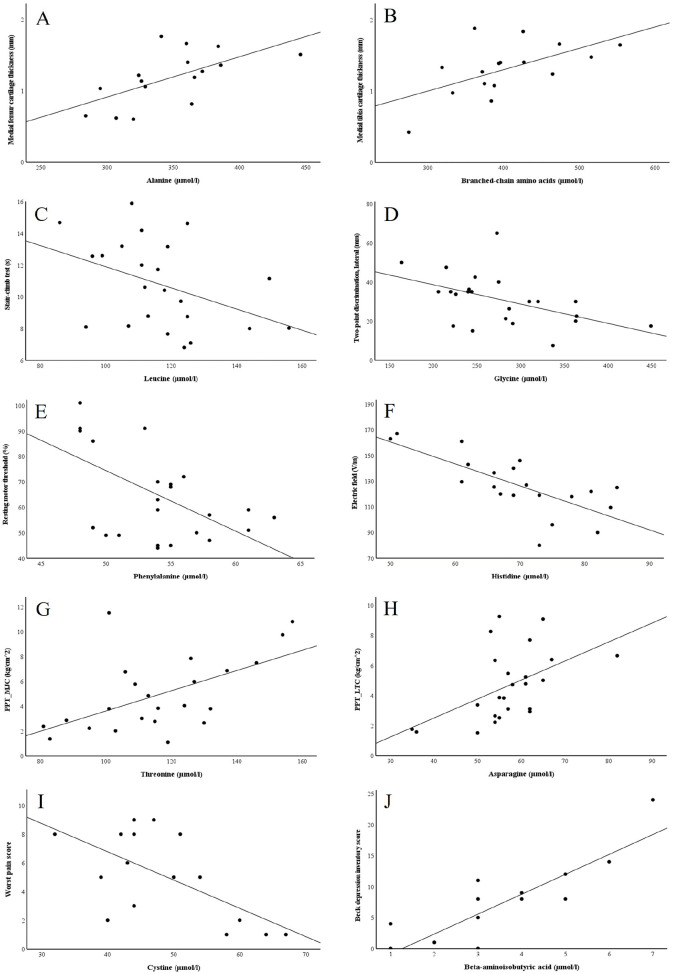
Scatter plots depicting the interrelationships between selected plasma amino acid variables and **(A–B)** cartilage thickness, **(C)** physical performance, **(D)** two-point discrimination threshold, **(E–F)** neuromuscular measurements, **(G–I)** pain parameters, and **(J)** Beck depression inventory in controls and knee osteoarthritis patients. PPT = pressure pain threshold; MJC = medial joint capsule; LTC = lateral tibial condyle.

To assess the interactions of individual AAs with knee pain and function, we performed a systematic analysis of Spearman correlations between all variables, across the controls and KOA patients (baseline and 12 months post-surgery). Several statistically significant correlations were observed between AA concentrations and cartilage loss, pain, stiffness, functional impairment, and mental health (data not shown). With the univariate ANOVA, we further tested the most relevant of these associations adjusted for the potential confounding factors, age and BMI. The significant associations are presented in Supplemental **Table S2**, and a selection of them is shown in **Figure 3**. For instance, higher PSer levels were associated with less morning stiffness, PSer and cystine with smaller pain symptoms, Asn with lower pain sensitivity, and BCAAs with thicker articular cartilage, better stair-climb performance, and lower rMTs. Thicker cartilage was also linked to elevated Ala and Met concentrations, and lower rMTs with higher Met, Phe, and Pro levels. Elevated LICI-likelihood was connected to higher *α*-aminobutyric acid (AABA) concentrations. Phe, Thr, and 3-MetHis were associated with lower pain sensitivity, while Bala, Asp, and AAA were linked to longer duration of knee pain, worse pain symptoms, or higher pain sensitivity. Gly, His, Cit, BAIBA, and Phe were connected to better TPD or higher thresholds for warm detection or heat pain. Regarding mental health, BAIBA and HLys were positively associated with BDI and BAI, whereas AABA and 3-MetHis had inverse associations with BAI.

### Pathway Analysis

**Figure 4.**
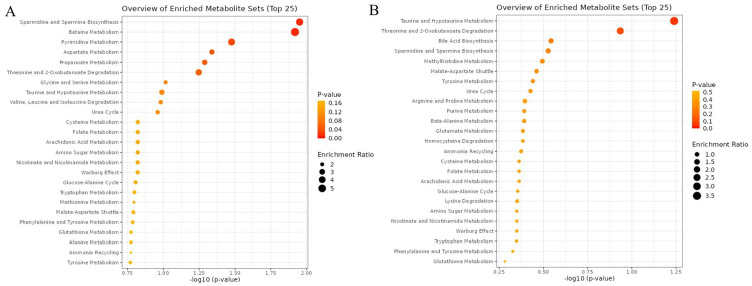
Enriched metabolite sets in plasma amino acids of **(A)** baseline knee OA (KOA) and of **(B)** post-surgery (12 months) KOA obtained by MetaboAnalyst.

According to the metabolite set enrichment analysis, spermidine and spermine biosynthesis, betaine metabolism, pyrimidine metabolism, and aspartate metabolism were particularly disturbed in baseline KOA (**Fig. 4A**), while taurine and hypotaurine metabolism was affected in post-surgery KOA (**Fig. 4B**).

**Figure 5.**
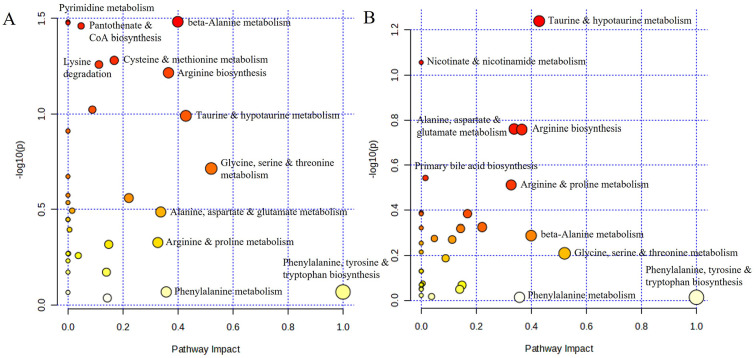
Summary of metabolic pathway analysis by using MetaboAnalyst in **(A)** baseline knee OA (KOA) and **(B)** post-surgery (12 months) KOA. The *P* value (circle color) and pathway impact value (circle size) were calculated from the pathway enrichment analysis and pathway topology analysis, respectively.

In the metabolic pathway analysis, *β*-alanine metabolism, pyrimidine metabolism, pantothenate and CoA biosynthesis, cysteine and methionine metabolism, lysine degradation, arginine biosynthesis, taurine and hypotaurine metabolism, phenylalanine, tyrosine, and tryptophan biosynthesis, and glycine, serine, and threonine metabolism displayed the highest statistical significance and/or pathway impact for baseline KOA (**Fig. 5A**). Regarding post-surgery KOA, taurine and hypotaurine metabolism, nicotinate and nicotinamide metabolism, alanine, aspartate, and glutamate metabolism, arginine biosynthesis, primary bile acid biosynthesis, arginine and proline metabolism, phenylalanine, tyrosine, and tryptophan biosynthesis, glycine, serine, and threonine metabolism, and *β*-alanine metabolism had the highest statistical significance and/or pathway impact (**Fig. 5B**).

**Figure 6.**
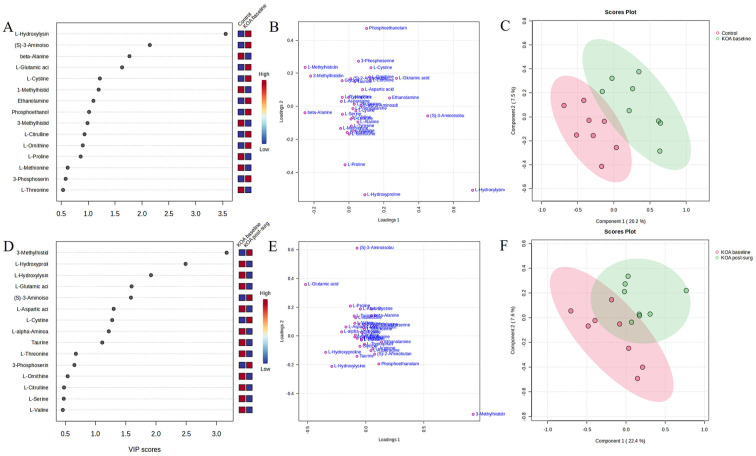
Amino acids (AAs) and related compounds associated with metabolic pathways in knee osteoarthritis (KOA). **(A, D)** Variable importance in projection (VIP) scores, **(B, E)** loadings plots of individual AAs, **(C, F)** partial least squares discriminant analysis scores plots. Panels **(A–C)** show baseline KOA and panels **(D–F)** post-surgery (12 months) KOA.

Plasma HLys, BAIBA, Bala, Glu, cystine, 1-MetHis, and ethanolamine showed the highest VIP scores for baseline KOA (**Fig. 6A**), and 3-MetHis, HPro, HLys, Glu, BAIBA, Asp, and cystine for post-surgery KOA (**Fig. 6D**). In PLS-DA, components 1–2 separated well the control and KOA baseline samples (**Fig. 6C**), while the separation was less satisfactory for the baseline and post-surgery KOA samples (**Fig. 6F**).

**Figure 7.**
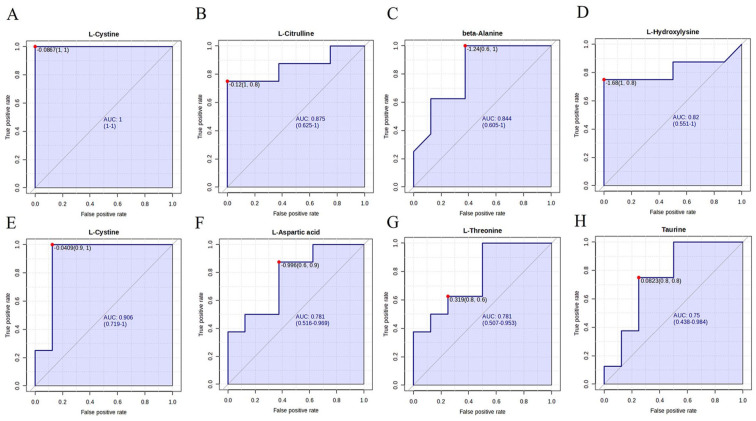
Receiver operating characteristic analysis of selected amino acids and related compounds in **(A–D)** knee osteoarthritis (KOA) patients and in **(E–H)** KOA patients after knee replacement surgery, AUC = area under the curve.

The ROC analysis for baseline KOA showed that plasma cystine had the greatest diagnostic potential with the AUC of 1.000 and the sensitivity and specificity of 1.000 (**Fig. 7A**, Table 1). For the next best metabolites (Cit, Bala, and HLys), the AUC values were 0.820–0.875, and the sensitivity and specificity were 0.750–1.000 and 0.625–1.000, respectively (**Fig. 7B-D**). Cystine also had the greatest diagnostic value in post-surgery KOA, with the AUC of 0.906, sensitivity of 1.000, and specificity of 0.875 (**Fig. 7E**). Asp, Thr, and Tau were the next 3 metabolites with the highest diagnostic potential (AUC 0.750–781, sensitivity 0.625–0.875, specificity 0.625–0.750; **Fig. 7F-H**).

**Table 1.** AUC, Sensitivity, and Specificity for Selected Amino Acids to Identify KOA Patients.

Comparison	Biomarkers	AUC	Sensitivity	Specificity
Baseline KOA *vs.* control	Cystine	1.000	1.000	1.000
	Citrulline	0.875	0.750	1.000
	*β*-Alanine	0.844	1.000	0.625
	Hydroxylysine	0.820	0.750	1.000
Post-surgery KOA *vs*.	Cystine	0.906	1.000	0.875
baseline KOA	Aspartate	0.781	0.875	0.625
	Threonine	0.781	0.625	0.750
	Taurine	0.750	0.750	0.750

AUC = area under the curve; KOA = knee osteoarthritis.

In the Random Forest analysis, one KOA patient was misclassified among controls (data not shown) and, thus, the classification error for controls was 0 and for baseline KOA 0.125 (out-of-bag error 0.0625). Cystine, Cit, Met, HLys, Ile, Orn, and Bala had the highest mean decrease accuracies. The classification was less accurate for baseline and post-surgery KOA, where the classification errors for both groups and the out-of-bag error were 0.375. Cystine, Orn, Tau, Thr, and Asp showed the highest mean decrease accuracies in this case.

**Figure 8.**
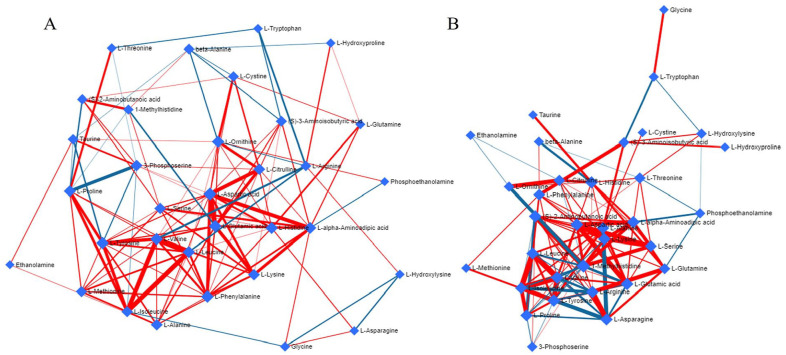
Correlations of metabolic pathways of amino acids and related compounds **(A)** in knee osteoarthritis and **(B)** after function restorative surgery. Blue connection lines represent negative correlations, whereas red connection lines represent positive correlations. Blue diamonds represent metabolites linked to pathways related to the input metabolites, forming clusters. The thicker the connection line, the stronger the relationship between the metabolites.

Correlations of metabolic pathways in baseline KOA compared with controls, and in the functional restoration of the knee joint compared with the preoperative situation, can be observed in **Figures 8A-B** as interactive networks.

## Discussion

The present study investigated associations between cross-sectional and longitudinal information from systemic AA profiles and subjective and objective assessment of pain, physical performance, neuromuscular function, and mental health in KOA. There were significant differences in the AA concentrations between the study groups, and we found perturbations of several key metabolic pathways in KOA and significant associations between AAs and other measured parameters. The main findings of the study were as follows: (1) plasma Asp, Bala, and AAA were positively linked to KOA pain, while Asn, cystine, and PSer inversely associated with pain parameters. (2) Several plasma AAs, such as His, Tau, Leu, and Met, may modulate corticospinal excitability, and (3) plasma Ala and several EAAs were positively associated with articular cartilage thickness. In addition, (4) BAIBA, HLys, AABA, and 3-MetHis could be connected to psychological well-being. (5) Several putative biomarkers could be identified to associate with KOA, of which cystine was among the most significant. (6) Pyrimidine metabolism, *β*-alanine metabolism, and phenylalanine, tyrosine, and tryptophan biosynthesis were among the top metabolic pathways disturbed in KOA. (7) Joint replacement surgery reduced pain and improved joint function, but the effects of surgery on plasma AA levels were less obvious.

**Figure 9.**
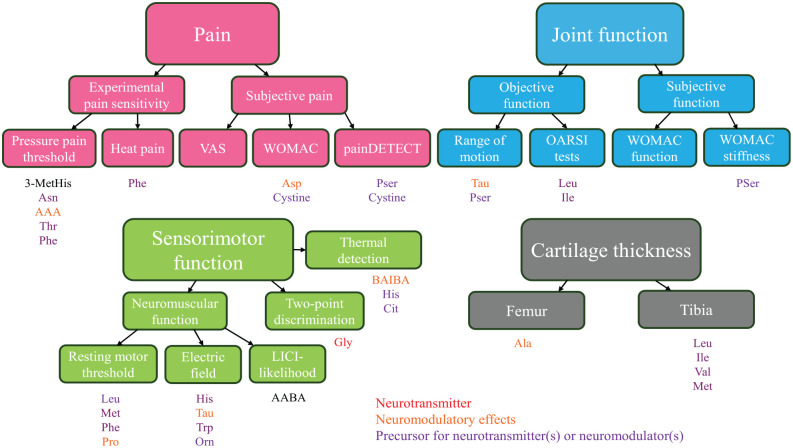
A visual summary of amino acid (AA) predictors for pain, physical and sensorimotor functions, and articular cartilage thickness. Individual AAs that best explained the variability in the measured parameters based on the univariate analysis of variance (*P* < 0.05) are presented with different font colors as indicated. AAs with red font color can be considered to act as neurotransmitters, those with orange font color may have neuromodulatory properties, and those with purple font color can be regarded as direct, or in some cases, indirect precursors for neurotransmitters or neuromodulators.^7,33-36^ LICI = long-interval cortical inhibition; OARSI = Osteoarthritis Research Society International; VAS = visual analog scale; WOMAC = Western Ontario and McMaster Universities Osteoarthritis Index.

Both baseline and post-surgery KOA were featured with alterations in alanine, aspartate, and glutamate metabolism pathway. Plasma Ala concentrations were positively associated with medial femur cartilage thickness, and several EAAs, including Met and BCAAs, with medial tibia cartilage thickness (**Fig. 9**). Except of Ala, these AAs are not among the most abundant AA components of cartilage^29^ unlike, for instance, Gly and Pro that are important constituents of collagen.^30^ It is plausible that low availability of particular AAs could contribute to insufficient collagen synthesis and lead to cartilage damage in OA, as increased concentrations of Gly, Pro, and lysine (Lys) in culture medium have been documented to enhance collagen synthesis by chondrocytes.^31^ In addition, orally administered collagen can reduce the symptoms of OA by improving total WOMAC and VAS scores.^32^ Decreased Ala concentrations in OA cartilage may be associated with the degradation of the collagen framework with OA progression.^11^

The present study documented positive associations of plasma Asp, Bala, and AAA concentrations to KOA pain (**Fig. 9**). Asp is closely linked to the TCA and urea cycles^33^ and, previously, its levels in SF increased in patients with synovitis.^6^ It has been proposed to serve as the secondary excitatory neurotransmitter in the CNS and to be involved in the transmission of nociceptive information from the spinal cord to higher brain centers. In pathological states, excitatory AAs have been suggested to be released in excess into SF and to induce local inflammation and pain. When administered into the joint with Glu and Arg, Asp produces hyperalgesic and allodynic responses, while the administration of Glu receptor antagonists reverses these changes.^15^ Bala, which is synthesized by pyrimidine catabolism and is also a degradation product of the dipeptides carnosine and anserine, acts as an agonist of both Gly and GABA_A_ receptors and is therefore a potential inhibitory neurotransmitter in the CNS.^37^ Additionally, AAA has been demonstrated to have neurological effects. It is a Glu analogue produced by the degradation of Lys, a potential marker of type 2 diabetes risk, and an astroglia-specific toxin associated with depression-like behavior.^38,39^ In lipopolysaccharide-inflamed mice, AAA can antagonize antinociception by the MMG22 ligand effective in the treatment of neuropathic pain.^40^ Our findings were reflected in the pathway analysis, showing that pyrimidine metabolism, *β*-alanine metabolism, pantothenate and CoA biosynthesis, lysine degradation, and alanine, aspartate, and glutamate metabolism were among the most disturbed pathways in KOA.

Several measured variables, including Asn, cystine, and 3-MetHis, were inversely associated with different pain parameters (**Fig. 9**). Asn is a precursor to Asp, and deficiencies in its synthesis can lead to structural abnormalities in the brain and cognitive deficits.^7^ Asn in SF and urine previously decreased in OA animal models,^41^ and its plasma levels showed an inverse correlation with VAS global well-being in RA patients.^14^ According to Wang *et al.*,^42^ N-*α*-acetyl-l-asparagine that can be produced from Asn was significantly associated with severe KOA and could be a useful serum biomarker for predicting KOA severity. Cysteine (Cys) in the blood is typically in the form of cystine,^33^ which consists of 2 Cys molecules.^43^ Cys is a critical AA for T cell functions, as they lack the enzyme that converts Met to Cys.^44^ It serves as an excitatory neurotransmitter and a precursor for Tau.^7^ As Cys is a building block to glutathione, the main antioxidant of the body, it could have potential in OA therapies, and the observed negative association between cystine and pain supports this. Cys could have several applications in pain- and inflammation-related disorders and, for instance, it has been observed in a murine model that Cys prodrugs can provide protection against colitis.^45^ In the present study, cysteine and methionine metabolism was among the disturbed pathways in baseline KOA, and cystine showed the greatest AUC, sensitivity, and specificity in both baseline and post-surgery KOA. Our findings agree with Sasaki *et al.*,^43^ who have previously suggested high cystine levels as a key biomarker for identifying patients at high risk of KOA progression caused by synovitis.

According to the pathway analysis, phenylalanine, tyrosine, and tryptophan biosynthesis and glycine, serine, and threonine metabolism were altered by KOA. Thr, Phe, and Pser were observed to be negatively associated with pain sensitivity or pain symptoms (**Fig. 9**). Thr and Phe are precursors for particular neurotransmitters (Gly, catecholamines via tyrosine [Tyr]),^7^ which could partly explain their connections to pain parameters. PSer is a precursor for Ser synthesis, and it is also produced by a post-translational phosphorylation of Ser residues in proteins, and increased PSer levels may derive from the hydrolysis of phosphoproteins and phosphatidylserine.^34^ Previous literature on the connections of PSer to pain is limited, but plasma Ser was shown to have an inverse correlation with VAS global well-being in RA patients,^14^ and a combination of 6 non-essential AAs (NEAAs), including Ser, decreased VAS pain scores in adults with joint discomfort but no diagnosed joint disorder.^46^ In addition to painDETECT scores, the present study also observed an inverse association between PSer levels and morning stiffness in the knee joint.

Regarding physical function tests, higher plasma BCAA concentrations improved the performance in the stair-climb test (**Fig. 9**), and there was also a positive correlation between BCAAs and walking speed, but it did not remain significant in the univariate ANOVA adjusted for age and BMI. BCAA supplements are marketed for muscle growth, improved physical performance, and reduced exercise-induced fatigue and, according to current literature, they may have beneficial influence on muscle mass, strength, and soreness, while effects on performance appear to be negligible.^47,48^ Paradoxically, BCAAs may also promote oxidative stress and proinflammatory status as documented in peripheral blood mononuclear cells.^49^ BCAAs are not produced by the body but must be obtained from the diet to be oxidized in skeletal muscles. In addition to physical performance, the present study also documented positive associations of BCAAs with corticospinal excitability (rMT) and tibial cartilage thickness. Regarding NEAAs, oral supplementation with a combination of Ala, Asp, Glu, Gly, Ser, and Pro has previously reduced the difficulty in performing daily activities in participants with joint discomfort but no diagnosed joint disorder.^46^ Of these NEAAs, plasma Pro correlated with faster walking speed and Asp with physical disabilities in the present study, but these associations did not remain significant in the univariate ANOVAs adjusted for age and BMI.

Based on neuromuscular testing with nTMS, high Met, Phe, Pro, and BCAA concentrations were connected to decreased rMTs and, in addition, elevated His, Tau, Orn, tryptophan (Trp), TAAs, and NEAAs reduced the electric field strength in the brain cortex (**Fig. 9**). The observed associations suggest that plasma levels of these AAs, especially His, may affect corticospinal excitability.^50^ Tau can act as an agonist at Gly and GABA_A_ receptors in the brain, and it was previously found to decrease cortical excitability and to exert antiseizure effects through its agonist activity at extrasynaptic GABA_A_ receptors containing the δ subunit.^51^ It can also induce larger MEPs after a non-fatiguing exercise. Taurine and hypotaurine metabolism emerged as a significantly altered metabolic pathway for both baseline and post-surgery KOA in the present study. Tau may hold potential for drug design to treat pathologies, such as depression, epilepsy, and alcoholism, through GABA_A_ receptors,^52^ but with a lower risk of harmful side effects. In addition, AABA was linked to LICI-likelihood and may, thus, be a modulator of GABA_B_ receptor inhibition.^53^ A moderate increase in LICI has been previously observed in people with pain,^54^ raising the hypothesis that intracortical inhibition would be elevated in chronic pain conditions and could also be associated with the observations on rMT.

Higher plasma Gly levels were noted to be associated with better tactile acuity, that is, lower TPD thresholds (**Fig. 9**). Gly is the main inhibitory neurotransmitter in the spinal cord, brain stem, and cerebellum^7^ and a constituent of proteoglycans in articular cartilage.^55^ Glycine transporter 1 can be involved in pain perception and movement, and its dysfunction has been implicated in neuropathic pain.^7^ His, Cit, and BAIBA increased with the warm detection threshold and Phe with the heat pain threshold in the present study. Plasma Glu has been shown to correlate with the warm detection threshold in patients with complex regional pain syndrome.^56^ However, previous knowledge about the connections of other AAs to thermal sensitivity remains limited.

Previous studies have demonstrated links between AA concentrations and mental health. This is expected, given that some AAs directly serve as neurotransmitters or their precursors. Deficiencies in AAs, such as Trp and Tyr, can lead to depression due to decreased concentrations of serotonin and dopamine, respectively,^57^ and circulating AAs, including Glu, Asp, and Gly, are potential diagnostic biomarkers for depression.^58^ In the present study, plasma BAIBA and HLys concentrations were documented to increase with BDI and BAI scores, while AABA and 3-MetHis may have protective effects. Previous literature on the connections of these compounds to mental well-being is scarce. Of the listed AAs, BAIBA is a contraction-induced myokine with antioxidative and anti-inflammatory effects,^59^ and its plasma levels increase with exercise.^60^ It is produced by pyrimidine degradation and by the catabolism of Val, and may function as a partial agonist of the Gly receptor with potential effects on its activation and the efficacy of GABA.^37^ In agreement, pyrimidine metabolism emerged among the most disturbed pathways in KOA, and BAIBA showed high VIP scores suggesting diagnostic value. A previous study reported reduced BAIBA concentrations in the plasma of patients with major depressive disorder.^61^ HLys, which was found to have clinically relevant potential for baseline and post-surgery KOA, is characteristic of collagen and collagen-like proteins, produced by a post-translational modification of Lys, and crucial for collagen glycosylation and crosslinking.^62^ AABA has been previously suggested as a potential marker for depression in older adults, with reduced plasma levels in the depressive group,^63^ corroborating the findings of the current study. Additionally, 3-MetHis emerged as a potential biomarker, particularly for the metabolic state of post-surgery KOA. It is post-translationally modified from His, excreted in urine, and can be utilized as an indicator of myofibrillar protein degradation.^64^

In the present study, only minor differences in plasma AA concentrations could be demonstrated between the KOA patients and healthy controls, and the findings contrast with a recent systematic review and meta-analysis by Liao *et al.*^65^ They reported elevated serum levels of Trp, Lys, and Leu and decreased concentrations of Pro, Phe, Gln, Cit, and Asn in OA patients. The discrepancy between our results and the cited literature could be attributed to differences in sample size, patient characteristics (e.g., disease severity, comorbidities), analytical platforms, or statistical power. It is also possible that metabolic alterations in OA are context-dependent and may not manifest uniformly across all cohorts. These findings highlight the need for further research to clarify the consistency and potential causality of AA changes in OA, as the present study explored regressions and not direct causal associations between AA levels and KOA risk. However, the positive associations of plasma Ala and Ile levels with cartilage thickness support recent findings of Cui *et al.*,^66^ which suggest that higher Ala and Ile concentrations in serum may reduce OA risk, particularly in the knee and hip joints.

Study limitations also include the small sample size and the fact that it was not possible to match the controls and KOA patients for age and BMI, which is unfortunately very often the case in KOA studies. However, by using the univariate ANOVA adjusted for these confounding factors, we were able to assess the latter issue, and several significant associations remained even after controlling for the confounders. The possibility of type II errors (i.e., failing to detect significant differences between experimental groups) remains a concern due to the relatively small sample size. However, the analyses still identified clearly distinguishing AAs and metabolic pathways, demonstrating adequate statistical power for this type of screening study, which is intended to be followed by large-scale experiments. Although TKA improved self-reported pain, joint stiffness, and physical limitations of the KOA patients, the ROM of the affected joint, PPT on lateral tibial condyle, and physical function test results remained lower compared with the controls after 12 months. This indicates that the function of the OA knee did not return to the level of the healthy joint, or the required recovery period would have been longer than 12 months, which could also have been reflected in the circulating AA concentrations. However, Hylkema *et al.*^67^ previously reported that in working-age patients, physical impairments and activity limitations mainly improved during the first 3 months after TKA, with no substantial improvements between 12 and 24 months. This suggests that a longer follow-up period would probably have been unnecessary. The present results were obtained from end-stage KOA samples, which may limit the outreach of the conclusions to the wider population with milder forms of the disease. However, it is reasonable to assume that the biochemical changes leading to cartilage destruction would also start to appear at the early stages of the disease. Therefore, extrapolating from end-stage KOA findings could provide a viable starting point for studying the onset of the condition. It should also be borne in mind that the easily accessible blood plasma does not necessarily reflect the SF AA values, especially during non-symptomatic disease.

To conclude, KOA presents an important clinical problem for the aging population due to the frequency of occurrence, difficulty of early diagnosis, and lack of efficient cartilage-restoring therapies. AAs have emerged as potential biomarkers to predict KOA and as therapeutic targets for KOA pathogenesis and pain. The most significant effects of AAs on KOA symptomatology could be their functions as neuromodulators, along with the potential antioxidative and anti- and proinflammatory nature of particular AAs. The present study demonstrated significant associations between several plasma AAs and articular cartilage thickness, KOA pain, physical function, corticospinal excitability, and mental health, independent of age and body adiposity. Pyrimidine metabolism, *β*-alanine metabolism, cysteine and methionine metabolism, taurine and hypotaurine metabolism, and phenylalanine, tyrosine, and tryptophan biosynthesis were significantly disturbed metabolic pathways among others. Of special interest for future studies would be cystine for its potential diagnostic and prognostic value, and BAIBA for the interconnectedness of KOA with mental health. While KOA represents a disease continuum with multiple contributing factors, these simple and easily measurable AAs have now emerged as attractive markers and translational targets for KOA diagnosis and for the potential prevention of cartilage damage and the ensuing psychological comorbidity.

## Supplemental Material

sj-docx-1-car-10.1177_19476035251360189 – Supplemental material for Plasma Amino Acids Reflect Cartilage Loss, Osteoarthritis Pain, Functional Disability, and Mental Health in a Longitudinal Study with Total Knee Replacement Supplemental material, sj-docx-1-car-10.1177_19476035251360189 for Plasma Amino Acids Reflect Cartilage Loss, Osteoarthritis Pain, Functional Disability, and Mental Health in a Longitudinal Study with Total Knee Replacement by Anne-Mari Mustonen, Laura Säisänen, Lauri Karttunen, Petro Julkunen, Amir Esrafilian, Jusa Reijonen, Jussi Mäki, Heikki Kröger, Jari Arokoski and Petteri Nieminen in CARTILAGE

sj-docx-2-car-10.1177_19476035251360189 – Supplemental material for Plasma Amino Acids Reflect Cartilage Loss, Osteoarthritis Pain, Functional Disability, and Mental Health in a Longitudinal Study with Total Knee Replacement Supplemental material, sj-docx-2-car-10.1177_19476035251360189 for Plasma Amino Acids Reflect Cartilage Loss, Osteoarthritis Pain, Functional Disability, and Mental Health in a Longitudinal Study with Total Knee Replacement by Anne-Mari Mustonen, Laura Säisänen, Lauri Karttunen, Petro Julkunen, Amir Esrafilian, Jusa Reijonen, Jussi Mäki, Heikki Kröger, Jari Arokoski and Petteri Nieminen in CARTILAGE
