# Supplementary material for: Plasma Amino Acids Reflect Cartilage Loss, Osteoarthritis Pain, Functional Disability, and Mental Health in a Longitudinal Study with Total Knee Replacement
Source: Cartilage. 2025 Aug 10;17(3):522–37. doi: 10.1177/19476035251360189 (PMC12339497; doi:10.1177/19476035251360189)
Supplement: Supplementary file 2 [file sj-docx-2-car-10.1177_19476035251360189.docx]

**Supplementary Table S2.** Univariate analyses of variance for measurements of cartilage thickness, physical medicine, and neuromuscular function explained by different amino acids (AAs) or related compounds in the plasma of controls and knee osteoarthritis patients. Only analyses that are statistically significant when adjusted for age and body mass index are reported.

| Dependent variable |  | Amino acid | R squared | F | *P* |
| --- | --- | --- | --- | --- | --- |
| *Cartilage thickness* | Medial tibia | Methionine | 0.484 | 5.160 | 0.042 |
|  |  | Valine | 0.495 | 5.535 | 0.037 |
|  |  | Isoleucine | 0.509 | 6.025 | 0.030 |
|  |  | Leucine | 0.565 | 8.347 | 0.014 |
|  |  | Branched-chain AAs | 0.530 | 6.834 | 0.023 |
|  |  | Essential AAs | 0.562 | 8.220 | 0.014 |
| Medial femur Alanine | | | 0.564 | 5.525 | 0.037 |
| *Physical function*  Extension | | Phosphoserine | 0.336 | 4.524 | 0.046 |
|  | | Taurine | 0.391 | 6.755 | 0.017 |
| Stair-climb | | Isoleucine | 0.478 | 4.753 | 0.041 |
|  | | Leucine | 0.502 | 5.956 | 0.024 |
|  | | Branched-chain AAs | 0.478 | 4.743 | 0.042 |
| Stiffness, in the morning | | Phosphoserine | 0.532 | 12.881 | 0.004 |
| *Quantitative sensory testing* | | | | | |
| Two-point discrimination (lat.) | | Glycine | 0.430 | 10.664 | 0.004 |
| Threshold for warm detection | | Histidine | 0.322 | 7.784 | 0.011 |
|  | | Citrulline | 0.315 | 7.517 | 0.013 |
|  | | *β*-Aminoisobutyric acid | 0.358 | 9.342 | 0.006 |
| Threshold for heat pain | | Phenylalanine | 0.453 | 14.222 | 0.001 |
| *Neuromuscular testing* | | | | | |
| Resting motor threshold | | Methionine | 0.523 | 6.670 | 0.018 |
|  | | Phenylalanine | 0.512 | 6.022 | 0.023 |
|  | | Proline | 0.483 | 4.599 | 0.044 |
|  | | Leucine | 0.555 | 8.558 | 0.008 |
|  | | Branched-chain AAs | 0.503 | 5.593 | 0.028 |
|  | | Essential AAs | 0.535 | 7.353 | 0.013 |
| Electric field | | Taurine | 0.428 | 11.013 | 0.004 |
|  | | Ornithine | 0.297 | 5.970 | 0.027 |
|  | | Histidine | 0.572 | 20.041 | <0.001 |
|  | | Tryptophan | 0.344 | 7.534 | 0.014 |
|  | | Non-essential AAs | 0.365 | 8.318 | 0.011 |
|  | | Total AAs | 0.346 | 7.614 | 0.014 |
| LICI-likelihood | | *α*-Aminobutyric acid | 0.562 | 5.358 | 0.041 |

*Pain parameters*

| PPT (med. tibial condyle) | Asparagine | 0.404 | 4.378 | 0.049 |
| --- | --- | --- | --- | --- |
|  | *α*-Aminoadipid acid | 0.435 | 5.746 | 0.026 |
| PPT (med. joint capsule) | Threonine | 0.383 | 5.377 | 0.032 |
|  | Asparagine | 0.395 | 5.840 | 0.026 |
|  | Phenylalanine | 0.383 | 5.353 | 0.032 |
| PPT (lat. tibial condyle) | Asparagine | 0.508 | 4.775 | 0.041 |
| PPT (lat. joint capsule) | 3-Methylhistidine | 0.539 | 9.551 | 0.006 |
| PainDETECT score | Phosphoserine | 0.535 | 9.199 | 0.010 |
| Pain, current | Phosphoserine | 0.507 | 12.025 | 0.005 |
| Pain, worst | Phosphoserine | 0.302 | 4.959 | 0.046 |
|  | Cystine | 0.408 | 7.989 | 0.015 |
| Pain, 30d | Phosphoserine | 0.435 | 8.493 | 0.013 |
| Pain, walking on a flat surface | Aspartic acid | 0.391 | 7.449 | 0.018 |
|  | Cystine | 0.395 | 7.567 | 0.018 |
| Pain, sitting or lying down | Cystine | 0.355 | 6.437 | 0.026 |
| Pain, standing | Cystine | 0.376 | 6.575 | 0.025 |
| Duration of knee pain | *β*-Alanine | 0.848 | 14.352 | 0.032 |
| *Mental health*  Beck depression inventory | *β*-Aminoisobutyric acid | 0.809 | 39.208 | <0.001 |
|  | Hydroxylysine | 0.581 | 11.309 | 0.006 |
| Beck anxiety inventory | *α*-Aminobutyric acid | 0.499 | 7.585 | 0.017 |
|  | *β*-Aminoisobutyric acid | 0.539 | 9.305 | 0.010 |
|  | Hydroxylysine | 0.528 | 8.792 | 0.012 |
|  | 3-Methylhistidine | 0.423 | 5.018 | 0.045 |

LICI = long-interval cortical inhibition, PPT = pressure pain threshold, lat. = lateral, med. = medial
