# Supplementary material for: Plasma Amino Acids Reflect Cartilage Loss, Osteoarthritis Pain, Functional Disability, and Mental Health in a Longitudinal Study with Total Knee Replacement
Source: Cartilage. 2025 Aug 10:19476035251360189. Online ahead of print. doi: 10.1177/19476035251360189 (PMC12339497; doi:10.1177/19476035251360189)
Supplement: sj-docx-1-car-10.1177_19476035251360189 – Supplemental material for Plasma Amino Acids Reflect Cartilage Loss, Osteoarthritis Pain, Functional Disability, and Mental Health in a Longitudinal Study with Total Knee Replacement [file sj-docx-1-car-10.1177_19476035251360189.docx]

**Supplementary Table S1.** Amino acid (AA) concentrations (μmol/l) in the plasma of controls and knee osteoarthritis (KOA) patients before and after joint replacement surgery (n = 8/group) (mean ± SE).

| **Amino acid** | **Control, baseline** | **KOA, baseline** | **KOA, 12 mo post-surgery** | ***p* group^a^** | ***p* sex^a^** | ***p* interaction^a^** |
| --- | --- | --- | --- | --- | --- | --- |
| Phosphoserine | 4.5 ± 0.19 | 5.1 ± 0.48 | 5.9 ± 0.40 | 0.001 | 0.063 | 0.063 |
| Taurine | 58.6 ± 1.35 | 65.8 ± 5.41 | 57.9 ± 2.75 | 0.094 | 0.140 | 0.310 |
| Phosphoethanolamine | 13.5 ± 1.84 | 20.0 ± 5.51 | 16.5 ± 2.48 | 0.032 | 0.005 | 0.208 |
| Aspartic acid | 4.5 ± 0.33 | 4.9 ± 0.23 | 4.3 ± 0.16 | 0.285 | 0.336 | 0.756 |
| Threonine | 132.6 ± 6.47 | 113.0 ± 8.05 | 107.5 ± 5.89 | 0.036 | 0.251 | 0.072 |
| Serine | 114.3 ± 5.58 | 100.6 ± 4.94 | 99.1 ± 4.80 | 0.023 | 0.455 | 0.628 |
| Asparagine | 61.4 ± 3.27 | 53.9 ± 3.24 | 55.5 ± 3.28 | 0.401 | 0.370 | 0.314 |
| Glutamic acid | 26.3 ± 3.55 | 40.9 ± 8.52 | 33.0 ± 5.25 | 0.494 | 0.130 | 0.402 |
| Glutamine | 551.6 ± 11.49 | 563.6 ± 31.99 | 581.6 ± 28.50 | 0.149 | 0.002 | 0.458 |
| Sarcosine | bdl | bdl | bdl |  |  |  |
| *α*-Aminoadipic acid | 3.1 ± 0.23 | 3.4 ± 0.32 | 3.0 ± 0.33 | 0.724 | 0.029 | 0.484 |
| Glycine | 261.4 ± 16.96 | 274.9 ± 24.98 | 284.8 ± 30.05 | 0.998 | 0.005 | 0.783 |
| Alanine | 351.4 ± 11.40 | 344.3 ± 17.51 | 383.6 ± 20.17 | 0.119 | 0.031 | 0.811 |
| Citrulline | 30.9 ± 2.02 | 37.5 ± 2.35 | 36.9 ± 2.05 | 0.003 | 0.169 | 0.153 |
| *α*-Aminobutyric acid | 20.1 ± 1.89 | 20.0 ± 2.00 | 19.8 ± 1.53 | 0.919 | 0.507 | 0.752 |
| Valine | 236.6 ± 15.02 | 221.9 ± 14.79 | 216.6 ± 7.14 | 0.587 | 0.006 | 0.493 |
| Cystine | 33.1 ± 2.45 | 42.8 ± 1.98 | 54.6 ± 3.35 | 1.058 × 10^–10^ | 0.041 | 0.793 |
| Saccharopine | bdl | bdl | bdl |  |  |  |
| Methionine | 24.4 ± 1.00 | 20.3 ± 1.13 | 21.4 ± 1.15 | 0.032 | 0.017 | 0.714 |
| Alloisoleucine | bdl | bdl | bdl |  |  |  |
| Cystathionine | bdl | bdl | 0.1 ± 0.13 | 0.657 | 0.509 | 0.657 |
| Isoleucine | 61.3 ± 3.68 | 53.9 ± 4.76 | 55.3 ± 3.18 | 0.610 | 0.000169 | 0.593 |
| Leucine | 122.8 ± 7.02 | 111.1 ± 7.39 | 112.1 ± 4.39 | 0.569 | 0.000002 | 0.453 |
| Argininosuccinate | bdl | bdl | bdl |  |  |  |
| Tyrosine | 56.5 ± 3.69 | 53.0 ± 3.98 | 54.8 ± 2.49 | 0.874 | 0.387 | 0.673 |
| *β*-Alanine | 3.0 ± 0.38 | 1.9 ± 0.23 | 2.1 ± 0.35 | 0.012 | 0.350 | 0.210 |
| Phenylalanine | 54.6 ± 1.48 | 52.4 ± 1.59 | 54.6 ± 1.67 | 0.496 | 0.000228 | 0.528 |
| *β*-Aminoisobutyric acid | 1.8 ± 0.41 | 2.9 ± 0.58 | 3.8 ± 0.67 | 0.003 | 0.239 | 0.114 |
| *γ*-Aminobutyric acid | bdl | bdl | bdl |  |  |  |
| Ethanolamine | 5.6 ± 1.27 | 6.3 ± 1.03 | 6.3 ± 0.73 | 0.473 | 0.066 | 0.161 |
| Hydroxylysine | 0.6 ± 0.18 | 1.6 ± 0.38 | 1.3 ± 0.31 | 0.004 | 0.078 | 0.466 |
| Ornithine | 59.8 ± 4.52 | 72.6 ± 6.05 | 69.9 ± 3.73 | 0.003 | 0.002 | 0.527 |
| Lysine | 173.1 ± 6.77 | 172.1 ± 4.83 | 177.4 ± 9.06 | 0.964 | 0.822 | 0.575 |
| 1-Methylhistidine | 16.5 ± 2.82 | 11.3 ± 1.54 | 12.1 ± 1.19 | 0.013 | 0.010 | 0.990 |
| Histidine | 70.9 ± 2.75 | 69.6 ± 3.54 | 70.1 ± 3.71 | 0.926 | 0.096 | 0.318 |
| Tryptophan | 53.6 ± 2.82 | 51.6 ± 2.95 | 52.5 ± 3.56 | 0.931 | 0.958 | 0.793 |
| 3-Methylhistidine | 3.6 ± 0.78 | 3.5 ± 0.98 | 4.1 ± 0.58 | 0.854 | 0.699 | 0.637 |
| Anserine | bdl | bdl | bdl |  |  |  |
| Carnosine | bdl | bdl | bdl |  |  |  |
| Arginine | 64.9 ± 5.98 | 61.1 ± 4.77 | 64.1 ± 3.79 | 0.949 | 0.144 | 0.201 |
| Hydroxyproline | 5.9 ± 1.43 | 6.0 ± 1.70 | 4.1 ± 0.83 | 0.216 | 0.006 | 0.379 |
| Proline | 178.5 ± 16.59 | 146.9 ± 24.20 | 158.4 ± 19.19 | 0.884 | 0.020 | 0.590 |
| Total AAs | 2861.1 ± 64.75 | 2810.4 ± 133.86 | 2884.9 ± 102.59 | 0.852 | 0.035 | 0.666 |
| Essential AAs | 929.9 ± 31.05 | 865.9 ± 42.46 | 867.5 ± 27.64 | 0.502 | 0.004 | 0.670 |
| Non-essential AAs | 1931.3 ± 39.68 | 1944.5 ± 95.58 | 2017.4 ± 76.31 | 0.446 | 0.092 | 0.565 |
| Branched-chain AAs | 420.6 ± 24.80 | 386.9 ± 26.41 | 384.0 ± 13.32 | 0.565 | 0.000313 | 0.485 |

^a^Generalized linear model, bdl = below detection limit
